# Cryopreservation toxicity and morphological outcomes in *Piaractus brachypomus* oocytes and embryos

**DOI:** 10.1590/1984-3143-AR2025-0080

**Published:** 2026-04-20

**Authors:** Melanie Digmayer, Darci Carlos Fornari, Lis Santos Marques, Jayme Aparecido Povh, Ricardo Pereira Ribeiro, Eduardo Thomé Nicoleti, Leonardo Queiroz Alencar, Louise Nex Spica, Tiantian Zhang, Danilo Pedro Streit

**Affiliations:** 1 Programa de Pós-graduação em Zootecnia, Universidade Estadual de Maringá – UEM, Maringá, PR, Brasil; 2 Grupo de Pesquisa Aquam, Programa de Pós-graduação em Zootecnia, Universidade Federal do Rio Grande do Sul – UFRGS, Porto Alegre, RS, Brasil; 3 Programa de Pós-graduação em Ciências Veterinárias, Universidade Federal do Rio Grande do Sul – UFRGS, Porto Alegre, RS, Brasil; 4 Centro de Modelos Biológicos Experimentais – CeMBE, Pontifícia Universidade Católica do Rio Grande do Sul – PUCRS, Porto Alegre, RS, Brasil; 5 Faculdade de Medicina Veterinária e Zootecnia – FAMEZ, Universidade Federal do Mato Grosso do Sul – UFMS, Campo Grande, MS, Brasil; 6 Faculty of Science and Technology, Bournemouth University, Poole, Dorset, United Kingdom

**Keywords:** embryo chilling sensitivity, cryopreservation toxicity, Low-rate freezzig, neotropical freshwater fish

## Abstract

This study evaluated the toxicity of cryoprotectants and the performance of low-rate freezing protocols for oocytes and embryos of *Piaractus brachypomus*, a Neotropical fish of increasing relevant for Brazilian aquaculture and genetic conservation. Eight cryoprotectant solutions based on methanol (MeOH) or dimethyl sulfoxide (Me_2_SO), combined with 0.25 M sucrose in L-15 or HBSS media, were tested for oocytes toxicity at 28 °C and post-freezing viability. In spite of histological and scanning electron microscopy (SEM) analyses indicated preservation of gross morphological features, none of the cryopreserved oocytes supported embryonic development, indicating loss of functional viability following cryoprotectant exposure and freezing. For embryos, two low-rate freezing protocols were evaluated: Protocol 1 (P1E), based on gradual cooling to −13 °C, and Protocol 2 (P2E), involving linear cooling to −60 °C followed by storage in liquid nitrogen. In P1E, eight treatments using 3.1 M MeOH combined with different concentrations of polyvinylpyrrolidone (PVP) or sucrose were tested. The highest proportion of morphologically viable embryos (15.3%) was obtained with MeOT + 0.45 M sucrose (SC5), which different significantly from the other treatments. In contrast, no morphologically viable embryos were recovered after P2E, likely due to inadequate dehydration and intracellular ice formation. Overall, high concentrations of permeant cryoprotectants and prolonged equilibration times were detrimental to oocyte and embryo viability, while sucrose showed better cryoprotective performance than PVP. Even though protocols tested were insufficient to ensure consistent post-thaw viability, the partial success observed in P1E under MeOH and sucrose combinations provides a relevant experimental basis for future refinement of conservation strategies and contributes to development of *ex situ* germoplasm conservation approaches for *P. brachypomus* and other Neotropical species.

## Introduction

Cryopreservation of fish gametes and embryos is a strategic tool for breeding programs and the conservation of genetic resources, particularly for species at risk of extinction. However, embryo cryopreservation in fish remains a significant challenge due to the large size, high water content, low membrane permeability, and extreme sensitivity to low temperatures exhibited by these cells (Zhang et al. 2006; Cabrita et al., 2006; Diwan et al., 2020). While significant challenges persist in fish embryo cryopreservation, emerging techniques have shown potential progress, exemplified by the partial success in zebrafish (*Danio rerio*) embryo cryopreservation combined with laser nanowarming (Khosla et al., 2020). Building on these advancements, oocyte cryopreservation has gained attention as a promising complementary approach, due to distinct cellular characteristics that may facilitate better preservation outcomes.

In this context, oocyte cryopreservation has been regarded as a promising alternative since the early 2000s for the preservation of the maternal genome, as oocytes present smaller cell volume and higher permeability compared to embryos (Isayeva et al., 2004). Despite the full viability of this technique still faces practical challenges, especially in non-model species, important progress has been made over the past decades, consolidating this approach as a relevant line of research in the fields of genetic conservation and aquaculture.

Studies using zebrafish have been conducted to advance the technique, with relevant outcomes reported for ovarian follicles (Tsai et al., 2009; Marques et al., 2015), ovarian tissue (Marques et al., 2019; Freitas et al., 2023), and oocytes at different developmental stages (Zampolla et al., 2009; Silva et al., 2018).

In neotropical species, most research has focused on *Piaractus mesopotamicus*, emphasizing embryo cryopreservation (Streit et al., 2007; Fornari et al., 2012, 2014; Neves et al., 2014; Lopes et al., 2011, 2014). On the other hand, Marques et al. (2018, 2021a) studied ovarian structures, in *P. mesopotamicus* and *Brycon orbignyanus*, respectively. Studies on oocyte cryopreservation in native South American species are scarce and, apart from a few works on *Steindachneridion parahybae* (Lopes et al., 2015, 2018, 2019), significant gaps remain regarding the application of the technique in roundfish species.

*Piaractus brachypomus* (pirapitinga) is a freshwater fish native to the Amazon and Orinoco River basins, with increasing economic importance in fish farming in Brazil and in several Asian countries (Guimarães and Martins, 2015; Kumar et al., 2018). Despite its productive and genetic relevance, no validated protocols currently exist for the cryopreservation of embryos or oocytes of this species.

To advance the development of such protocols, it is essential to understand the toxicity of cryoprotectants and the chilling sensitivity of oocytes and embryos. Compounds such as methanol (MeOT) and dimethyl sulfoxide (Me_2_SO) have been reported as effective due to their high permeability and low toxicity (Zhang et al., 2003, 2005; Seki et al., 2007, 2011). Additionally, the inclusion of non-permeating cryoprotectants like sucrose has shown protective effects (Xiao et al., 2008; Godoy et al., 2013; Marques et al., 2015).

Therefore, this study aimed to evaluate the toxic effects of cryoprotectants (MeOH and Me_2_SO combined with sucrose) on *P. brachypomus* oocytes and to test different cryopreservation protocols for embryos, with the goal of contributing to the *ex situ* genetic conservation of neotropical fish species.

## Methods

### Experiment side and animals

The study was conducted in a fish farm located in Rondônia, Brazil (11°41’46 0.95”S and 61°13’47 0.50”O). The broodstock fish used in the research were maintained in two ponds 800 m^2^ each, with a stocking density of 228 g of fish/m^2^. Five males and five females were used to obtain gametes, the reproduction method was induced spawning using carp pituitary extract (CPE), the average weight of male broodstock fish was 2.7 ± 1.2 and of female broodstock fish was 4.2 ± 0.9 kg. Fish were fed once daily 1% of body weight) a commercial diet with 32% crude protein and 3100 kcal digestible energy. Water quality parameters, mean temperature (29 ± 1 °C) and dissolved oxygen (5 mg/L ± 1.1), were measured daily.

### Ethics statement

All experimental procedures used in this study follow the rules edited by the “National Council for Animal Experimentation Control” of the Brazilian Ministry of Science, Technology and Innovation. Project approved by the local “Ethics Committee on Animal Use” provided by Brazilian legislation (number 172/2012).

### Oocyte collection

Four females (two for experiment 1 and two for experiment 2) were induced according to the recommendations of Vásquez-Torres (2005) to obtain oocytes. After 10 h of the second hormonal dose (290-310 Accumulated Thermal Unit - ATU) at 28 ± 2 °C, the females were manipulated and the oocytes were obtained through abdominal massage. Immediately after obtaining the oocytes, a pool was produced to carry out the tests described below.

### Design experiment 1. Toxicity and cryopreservation of *P. brachypomus* oocytes

#### Toxicity test of cryoprotectants on oocytes at room temperature (28 ± 2 °C)

**Figure 1:**
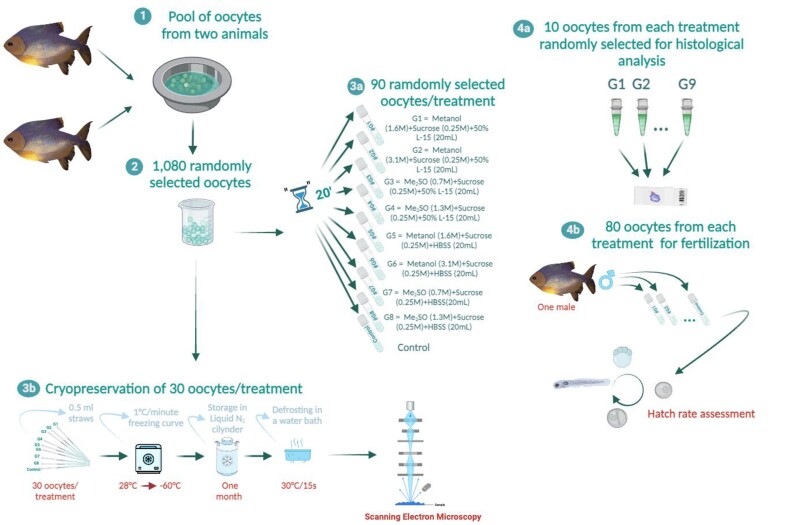
Infographic summarizing the development of the design of cryoprotectant toxicity tests and cryopreservation of *Piaractus brachypomus* oocytes.

One thousand and eighty oocytes (with eccentric nucleus, suitable for fertilization) were randomly separated (Figure 1 - steps 1 and 2). The number of oocytes per treatment was defined based on logistical constraints related to the induced spawning condition in the field and the evaluation of a significant number of samples per treatment to ensure homogeneity of the developmental stage. Oocytes were randomly distributed into nine groups, which were eight treatments and one control. The control treatment was composed of oocytes not exposed to any medium and cryoprotectants. Only oocytes that presented macroscopic characteristics compatible with fertilization (eccentric nucleus) were selected. The experiment was repeated three times and 90 oocytes were used in each treatment (Table 1; Figure 1 – step 3a).

**Table 1:** Cryoprotective solutions for *Piaractus brachypomus* oocytes for the toxicity test. Methanol (MeOH), Dimethyl sulfoxide (Me_2_SO), Sucrose (SUC), Leibovitz medium (L-15) and Hanks balanced salt solution (HBSS).

Experimental Treatment	Cryoprotectants (mol/L)			50% L-15 (q.s.p.)	HBSS (q.s.p.)
	MeOH	Me_2_SO	SUC		
G1	1.6	-	0.25	20 mL	-
G2	3.1	-	0.25	20 mL	-
G3	-	0.7	0.25	20 mL	-
G4	-	1.3	0.25	20 mL	-
G5	1.6	-	0.25	-	20 mL
G6	3.1	-	0.25	-	20 mL
G7	-	0.7	0.25	-	20 mL
G8	-	1.3	0.25	-	20 mL

To test the toxicity of cryoprotectant solutions, we used the equilibration time of 20 min (28 ± 2 °C) adapted from Plachinta et al. (2004), for embryos of native species (*P. mesopotamicus*) according to Streit (2005).

From the initial pool of 90 oocytes per treatment, 10 oocytes per each treatment were allocated for histological analysis (Figure 1 – step 4a). The remaining 80 oocytes from each treatment were fertilized with 30 μL of fresh milt obtained from a single male (Figure 1 – step 4b).

The hormonal induction protocol for semen collection followed the recommendations of Vásquez-Torres (2005). A volume of 0.1 mL of milt was collected for subjective evaluation of sperm motility and concentration. After checking for contamination according to Marques et al. (2021b), a sample of activated spermatozoa (2 μL) was obtained by diluting 20 μL of semen in 400 μL of distilled water and placing it on a slide for optical microscopy. It was then covered with a coverslip and immediately evaluated for sperm motility under optical microscopy at 400x magnification, with motility classified from 0 to 100%. To estimate sperm concentration, the samples were fixed in 10% buffered formaldehyde solution at a 1:1000 dilution (1 μL of semen: 999 μL of formaldehyde solution). An aliquot (10 μL) of the diluted semen was pipetted into each counting field of a Neubauer chamber covered with a coverslip, and allowed 15 min for cell stabilization. Using a microscope with 400x magnification and a manual counter, gametes were quantified by counting 10 squares. After cell counting, sperm concentration was calculated using the following equation (Sanches et al., 2011):


$$Spermatozoa\;mL-1\;=\;\left(\frac{\text{Σ}SPTZ}{10\;s.c}\right)\;x\;\left(\;\frac{25\;t.s\;x\;dilution\;x100}{\text{chamberdepth}\left(\text{mm}\right)}\right)\;$$ (1)

where: Spermatozoa mL^−1^: Number of spermatozoa per milliliter of sperm; ΣSPTZ: Total number of spermatozoa counted; 10 *s.c*: Squares counted; 25 *t.s*: Total squares; Dilution: Factor of dilution of the sperm by the fixative; Chamber depth: Normality 0.1 mm.

Based on the estimated motility (96%) and sperm concentration (17X10^9^ sperm/mL), a ratio of 120,000 sperm/oocytes, the selected oocytes were fertilized with 2.9 μL milt. The eggs were placed in 5 L conical incubator (28 ± 0.2 °C) for embryonic development until the stage selected for cryopreservation tests with embryos. Sperm motility was subjectively assessed with values of 96% and 17 × 10^9^ spermatozoa/mL, respectively. To ensure oocyte fertilization, a sperm:oocyte ratio of 120,000:1 was used according to Leite et al. (2013). In vitro incubation lasted 16 ± 0.5 h.

#### Slow rate freezing of oocytes

Thirty oocytes were selected based on macroscopic characteristics consistent with the eccentric nucleus stage, as previously reported from each treatment were exposed for 20 min to the cryoprotectant solutions (Table 1) and then placed into 0.5 mL straws (minitube for semen) (Figure 1 – step 3b). The cryopreservation curve used in the assays followed the protocol by Zhang et al. (1993). Samples, initially at room temperature of 29 ± 1 °C, were cooled at 1 °C/min using a controlled rate freezer (Dominium K, Biocom, Brazil) down to −60 °C, enucleation (moment that passes from liquid to solid state, ice) was performed at −5 at −7 °C, and then transferred to liquid nitrogen. After one month of storage in liquid nitrogen, the straws were heated in a water bath at 30 °C for 15 s, and the samples were then sent for scanning electron microscopy (SEM) analysis.

The following experiments are summarized in Figure 1.

### Design experiment 2. Cryopreservation of *P. brachypomus* embryos

In the second experiment, from the two selected females induced for reproduction, as described in *Oocyte collection*, a total of 5000 oocytes were fertilized using semen from a second male specimen. The semen collection methodology, as well as the evaluation of motility and obtaining the sperm concentration, followed the methodology described in *Experiment 1 - Cryoprotectant toxicity test in oocytes at room temperature (28 ± 2 °C)*. However, for this male, the motility obtained was 94% with a concentration of 18x10^9^ sperm/mL, using the same insemination dose of 120,000:1 sperm:oocyte. After the fertilization process, the eggs were distributed in a 5 L conical incubator (28 ± 0.2 °C) for embryonic development until the stage selected for cryopreservation tests.

#### Slow rate freezing Protocol 1 for embryos (P1E)

**Figure 2:**
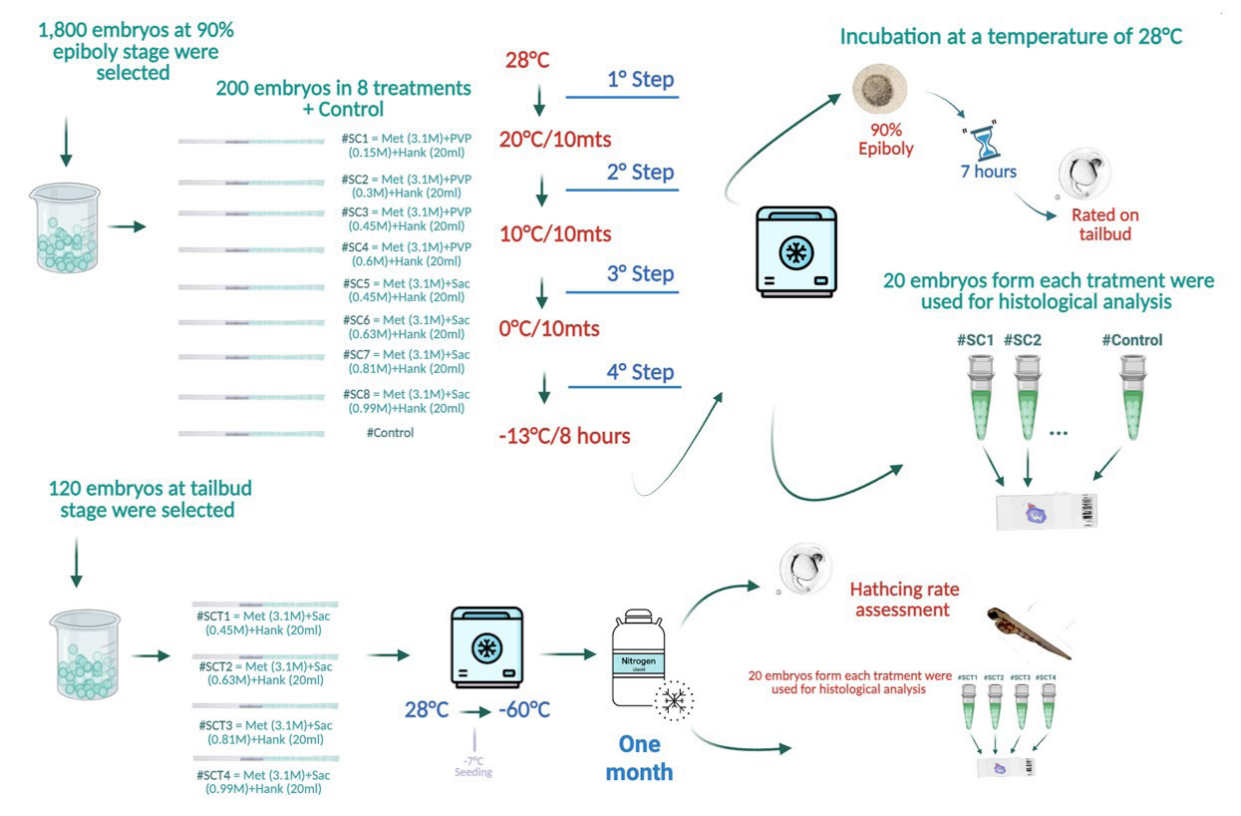
Infographic summarizing the development of the design of cryopreservation of *Piaractus brachypomus* embryos.

**Figure 3:**
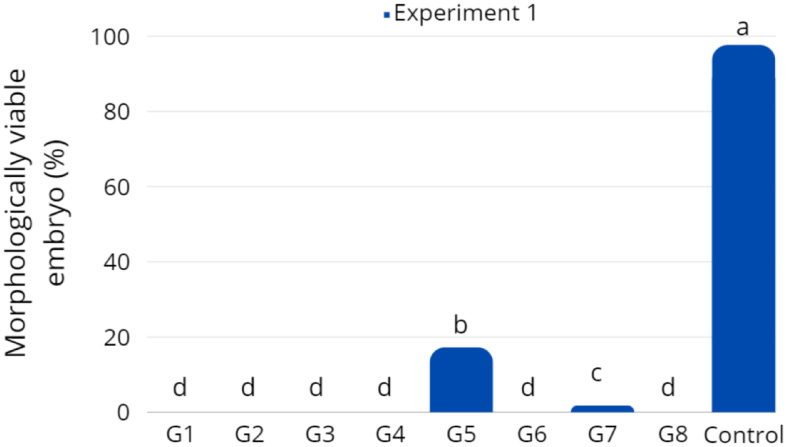
Rate morphologically viable embryo after hatching during 16 ± 0.5 hpf in Experiment 1 - *Piaractus brachypomus* oocytes exposed to the cryoprotectant solutions at room temperature (28 ± 2 °C). Bars with different superscript letters present statistical difference in significance level of 5% (*P* < 0.05).

Following incubation of 6 h at 28 ± 0.2 °C, 1,800 *P. brachypomus* viable embryos at 90% epiboly were selected by stereomicroscope (Table 2). Slow rate freezing of embryos was performed according to the cooling curve suggested by Lopes et al. (2011). Embryos were cooled from 28 ± 0.2 °C to 0 °C, keeping the temperature constant for 10 min every 10 °C drop. When the temperature reached 0 °C, the embryos were placed in a freezer at −13 °C for 8 h (Figure 2 – step 1). After the freezer storage period, the embryos were reheated, following the reverse of the cooling curve applied to the embryos, to 28 °C. Thus, the embryos, still at the 90% epiboly stage, were incubated for 8 ± 1 h until the larvae hatched at a temperature of 28 ± 0.2 °C in a 5 L conical incubator. Embryonic viability and morphological evaluation were performed at the end of organogenesis, pre-hatching of embryos, as morphologically described for *P. brachypomus* by Díaz-Olarte et al. (2010). Different embryonic stages (other than a differentiated pre-hatching embryo) have been classified as non-viable (Figure 2 – step 2a). From the 90% epiboly embryo samples that underwent the previously described heating curve, twenty embryos were sent for histological analysis (Figure 3 – step 2b). The experiment was repeated three times, and 200 embryos were used in each treatment, using 0.5 mL straws for filling and subsequent cooling.

**Table 2:** Cryoprotective solutions used in cryopreservation tests of *Piaractus brachypomus* embryos. Methanol (MeOH), polivinilpirrolidona (PVP), sucrose (SUC) and Hanks balanced salt solution (HBSS).

Experimental Treatment	Cryoprotectants (mol/L)			HBSS (q.s.p.)
	MeOH	PVP	SUC	
SC1	3.1	0.15	-	20 mL
SC2	3.1	0.30	-	20 mL
SC3	3.1	0.45	-	20 mL
SC4	3.1	0.60	-	20 mL
SC5	3.1	-	0.45	20 mL
SC6	3.1	-	0.63	20 mL
SC7	3.1	-	0.81	20 mL
SC8	3.1	-	0.99	20 mL
Control	-	-	-	20 mL

#### Slow rate freezing Protocol 2 for embryos (P2E)

*P. brachypomus* viable embryos, in tailbud stage (bud), were selected using the stereomicroscope and distributed into five treatments, which were four treatments and one control (Table 3). Embryos in experimental treatments were exposed for 20 min to the cryoprotectant solutions. Control treatment, embryos not exposed to cryoprotectants, was kept in HBSS. Afterward, embryos were loaded into 0.5 mL straws. Straws were placed into a controlled rate freezer (Dominium K, Biocom, Brazil) and cooled at 1 °C/min down to −60 °C, seeding was performed at −7 °C, and then immersed in liquid nitrogen. Samples were stored for approximately 30 days in liquid nitrogen (Figure 2 – step 3a). All treatments were cultured in a incubator at 28 ± 0.2 °C until hatching. Eight embryos from each treatment were selected and heated in a water bath at 30 °C for 15 s, followed by histological processing of the samples (Figure 2 – step 3b). The experiment was repeated three times, testing a total of 24 embryos in each treatment.

**Table 3:** Composition of cryoprotectant solutions for *Piaractus brachypomus* embryos used in Protocol 2 (P2E). Methanol (MeOH), sucrose (SUC) and Hanks balanced salt solution (HBSS).

Experimental Treatments	Cryoprotectants (mol/L)		HBSS (q.s.p.)
	MeOH	SUC	
SCT1	3.1	0.45	20 mL
SCT2	3.1	0.63	20 mL
SCT3	3.1	0.81	20 mL
SCT4	3.1	0.99	20 mL

The following experiments are summarized in Figure 2.

### Histological analysis

The selected samples in both experiments (1 and 2) were fixed with glutaraldehyde (2.5% solution in 0.1 M sodium phosphate buffer, pH 7.2). For processing, the samples were dehydrated in a series of alcohol solutions (70 to 100%) and embedded in resin blocks. The blocks were cut into 2 µm thick sections using a rotary microtome (Leica RM2245, Germany), and the slides were stained with hematoxylin-phloxine (Tolosa et al., 2003). Images were captured using a light microscope (Olympus BX 41) coupled to a high-resolution camera using the Q Capture Pro 51 software.

### Scanning Electron Microscopy (SEM)

For SEM analysis, samples containing 10 oocytes/treatment were fixed with glutaraldehyde (2.5% solution in 0.1 M sodium phosphate buffer, pH 7.2). Histological processing was the same as described above. For SEM analysis, samples were washed and post-fixed in osmium tetroxide 1% for 4 h. Then, samples were rewashed in buffer and subjected to dehydration in an ascending ethanol series. Drying was done with liquid CO_2_ followed by a coating of gold-palladium. Oocytes were examined and electronically micrographed by Scanning Electron Microscope JSM - 5410 (JEOL, USA).

### Statistical analysis

When working on oocytes and freezing embryos at −13 °C, the statistical differences between treatments, using model methodology generalized linear. To this end, estimating the probabilities of embryos developed for each treatment and comparing them, through the chi-square implemented in SAS Proc GENMOD. In the estimation process, the logit link function was used considering that the residues presented binomial distribution.

In the work on freezing embryos in liquid nitrogen, it was estimated that relative frequency of embryos preserved after thawing.

## Results

### Experiment 1. Toxicity and cryopreservation of *P. brachypomus* oocytes

#### Toxicity test of cryoprotectants on oocytes at room temperature (28 ± 2 °C)

The cryoprotectant solutions were toxic when the oocytes are exposed at 28 ± 2 °C. Embryo hatching rate were null in most treatments, except in G5 and G7, which showed survival rates of 17.3% and 1.8% respectively, after 16 ± 0.5 h of incubation (hpf). However, both were significantly lower compared to the control (97.7%) (*P* < 0.05; Figure 3).

**Figure 4:**
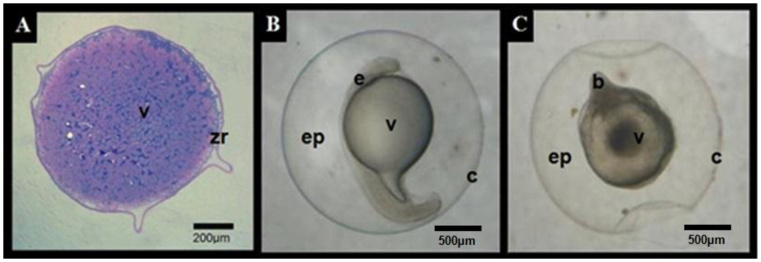
Histological analysis (hematoxylin-phloxine) of *Piaractus brachypomus* oocytes after toxicity test, 6 hpf (A). Developed morphologically embryo pre-hatching the final stage of the segmentation period with a free tail in 15-16 hpf (B) and non-viable embryo (90% stage of epiboly) (C). All the figures correspond following fertilization of *Piaractus brachypomus* oocytes exposed to the cryoprotectant solutions at room temperature (28 ± 2 °C). Yolk (v), zona radiate (zr), embryo (e), perivitelline space (ep), chorion (c) and blastoderm (b). This figure is in color in the electronic version.

After the toxicity test, histological sections at low magnification suggested preservation of gross, without allowing detailed assessment of subcellular organization (Figure 4A). Figure 4B shows a morphologically developed embryo (pre-hatching), whereas Figure 4C presents a non-viable embryo (90% stage of epiboly), obtained after exposure of oocytes to cryoprotective solutions at room temperature (28 ± 2 °C). Histological observations revealed qualitative morphological alterations in non-viable embryos revealed blastoderm disorganization, irregular yolk morphology and alterations in chorionic integrity.

#### Slow rate freezing of oocytes

**Figure 5:**
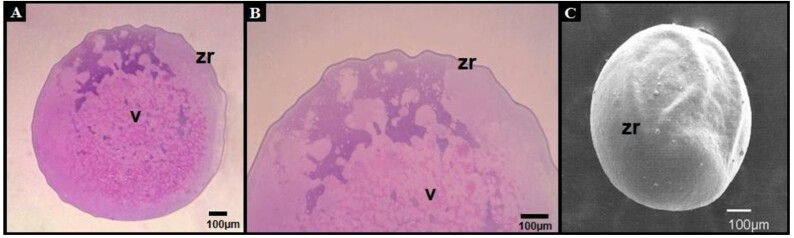
Histology *of Piaractus brachypomus* oocytes cryopreserved with 1.6M MeOT + 0.25M sucrose + Hank's solution (T5), cords with hematoxylinfloxin in (A, B) and in SEM (C). Yolk (v) and zona radiata (zr).

Histological (Figures 55B) and scanning electron microscopy (SEM; Figure 5C), these observations allowed for a qualitative assessment of the overall surface morphology of the oocytes. However, they did not provide sufficient resolution to support detailed ultrastructural interpretations.

### Experiment 2. Cryopreservation of *P. brachypomus* embryos

#### Slow rate freezing Protocol 1 (P1E) for embryos

**Figure 6:**
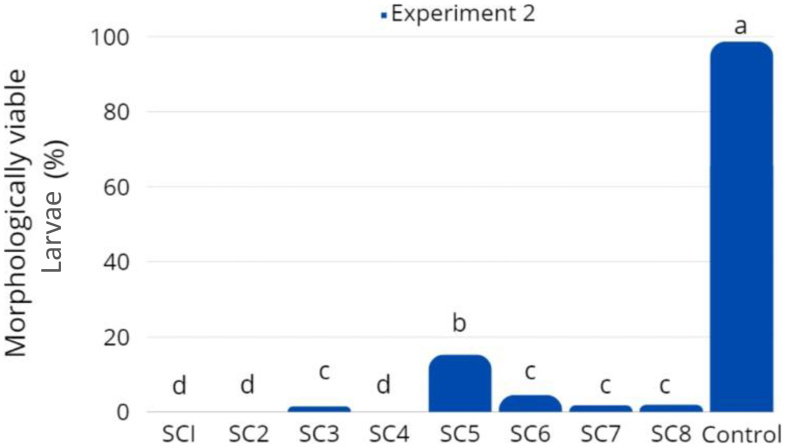
Morphologically viable *Piaractus brachypomus* larvae rate after 8 h in freezing −13 °C (P1E) from *Experiment 2*. Bars with different superscript letters present statistical difference in significance level of 5% (*P* < 0.05).

Among the treatments, the SC5 result in the highest morphologically viable embryos (15.3%; *P* < 0.05; Figure 6) after 15-16 hpf. Nonetheless, this rate was significantly lower than the the control (98.7%). There was no difference among treatments SC3 (1.5%), SC6 (4.6%), SC7 (1.8%), and SC8 (1.9%). In treatments SC1, SC2 and SC4, all embryos were non-viable (90% stage of epiboly).

For histological analysis of embryos at the 90% epiboly stage (the end of gastrulation) after 8 h at −13 °C in *Experiment 2*, treatments SC4, SC5 and SC8 were selected for histological analysis, along with the control. These treatments were selected to histologically characterize the embryos when subjected to incubation at a temperature of 28 °C to complete the embryonic cycle until larval hatching. Therefore, treatment SC4 represented non-viable embryos; SC5 had the highest viability; SC8 had low viability. Control embryos showed mostly intact morphology, though some exhibited disorganized blastoderm (Figures 77B). In SC5, embryos displayed intact chorion, well defined blastoderm involving the yolk and no visible cell damage (Figurse 7C and 7D). In contrast, SC6 showed disrupted blastoderm and chorion rupture with yolk extrusion (Figures 77F). Regarding the embryos treated as SC4, after warming, they exhibited morphological alterations and did not progress beyond the gastrulation stage, with no evidence of developmental advancement toward organogenesis. (Figures 77H).

**Figure 7:**
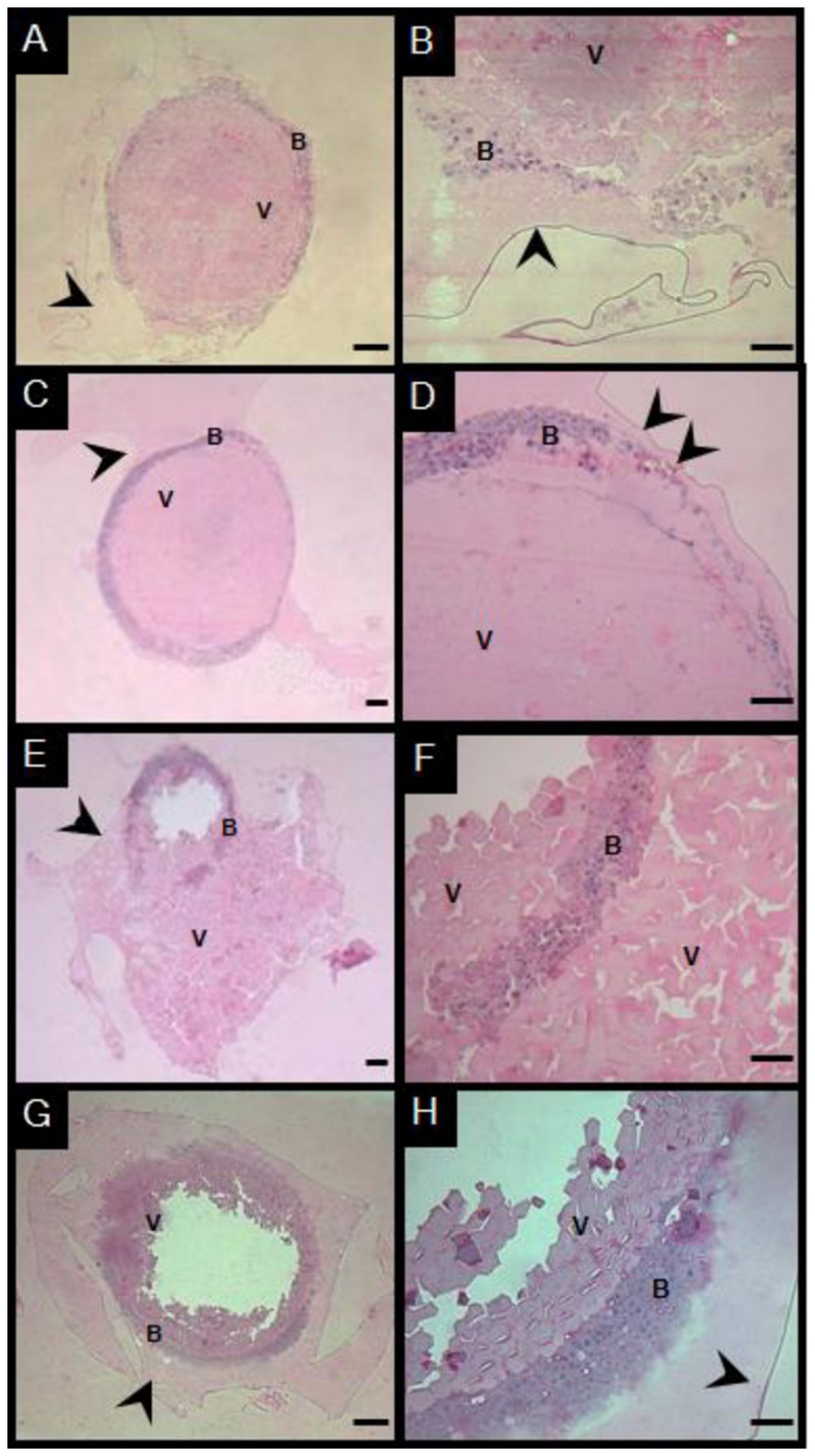
Photomicrograph of *P. brachypomus* embryos in 90% epiboly after 8 h in freezing −13 °C (P1E) from *Experiment 2* (A and B) and experimental SC5 (C and D), SC7 (E and F) and SC4 (G and H). Blastoderm (b), yolk (v) and chorion (arrow head). Scale bars: Figures A, C, E, G = 100µm; Figures B, D, F, H = 50µm. This figure is in color in the electronic version.

#### Slow rate freezing Protocol 2 (P2E) for embryos

There was no morphologically viable embryo after cryopreservation using P2E, wuile in the control group, 98% of the larvae hatched at 16 ± 0.5 hpf. Embryos classified as exhibiting apparent structural integrity immediately after warming were characterized by a well defined blastoderm, intact chorion and uniform and enclosed uniform yolk (Figures 88B). Treatments SCT1 and SCT4 showed a higher proportion of embryos with this apparent structural condition, while no embryos meeting these criteria were observed in treatments SCT2 and SCT3. Embryos from SCT2 and SCT3 exhibited pronounced morphological alterations, including irregularity shape, disorganized blastoderm and fusion of yolk granules (Figures 88D). Although most embryos from all treatments presented an intact chorion, this observation was limited to macroscopic morphological characteristics visible a low magnification. This apparent structural integrity was transient and did not result in progression to subsequent developmental stages.

**Figure 8:**
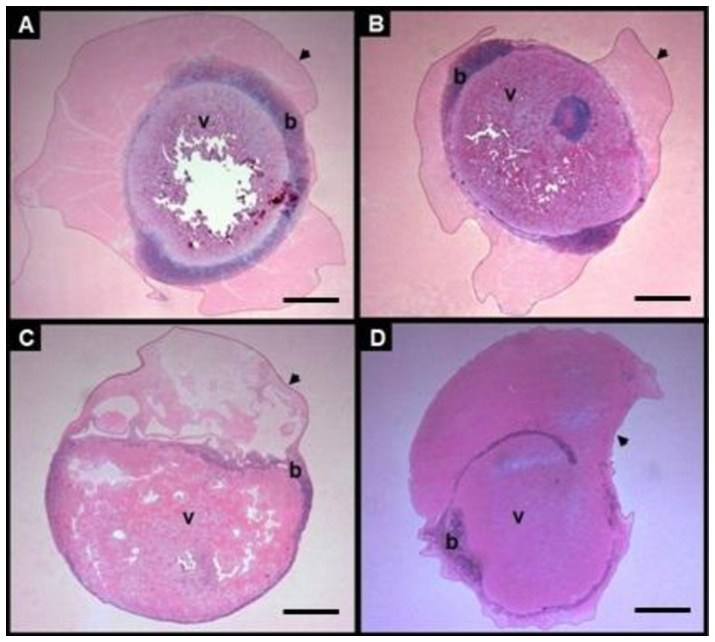
Photomicrograph of *Piaractus brachypomus* embryos after one month cryopreservation (P2E) from *Experiment 2*, illustrating general morphological characteristics observed at low magnification. Images A and B show embryos with apparent structural integrity immediately after thawing, characterized by a well defined blastoderm (b), intact chorion (arrow head) and uniform yolk (v). Images C and D show embryos with pronounced morphological alterations, including blastoderm disorganization and yolk granule fusion. Scale bars: 200µm. This figure is in color in the electronic version.

## Discussion

In the present study, cryopreservation was applied at the end of the gastrulation phase and no morphologically viable embryos were observed after thawing in any of the treatment tested. In spite of treatments SCT1 and SCT4 showed a higher proportion of embryos with apparent structural integrity immediately after thawing, this condition did not translate into functional viability or progression to subsequent development stages. In contrast, embryos from treatments SCT2 and SCT3 consistently showed pronounced morphological alterations, including blastoderm disorganization and yolk granule fusion. These observations indicate that macroscopic morphological appearance immediately after thawing is not a guarantee of embryonic developmental competence.

Although apparent morphological integrity of *P. brachypomus* oocytes was observed after cryoprotectant in some treatments, preservation of structure alone does not necessarily reflect normal hydration dynamics, membrane permeability or osmotic functionality. In the presente study, the hydration capacity of the oocytes was not directly measured, since the experimental design focused on evaluating the toxicity of the cryoprotectant and structural integrity and not on osmotic functionality. Similar response patterns have been reported for other Neotropical species, in which embryos maintained apparent morphological integrity after cooling or freezing, but failed to develop or hatch. For example, in *Prochilodus lineatus*, moderate cooling (−8 °C to −10 °C) resulted in hatching rates of up to 25-30%, while deep freezing resulted in complete loss of hatching (Ninhaus-Silveira et al., 2009; Machado et al., 2019). Similarly, *Piaractus mesopotamicus* embryos of showed partial hatching success (≈64%) after 12 h of controlled cooling at −8 °C (Fornari et al., 2012), while more advanced developmental stages suffered irreversible damage from cryopreservation (Lopes et al., 2011). These findings reinforce the idea that morphological preservation does not necessarily indicate biological functionality and highlight that post-thaw hatching are more reliable predictive indicator.

Histological and scanning electron microscopy analyses provided qualitative information on the overall morphological condition of oocytes and embryos after cryopreservation. At the magnification used, these techniques allowed visualization of general structural characteristics, such as blastoderm organization, chorion continuity and yolk morphology, but did not allow a detailed assessment of cellular or subcellular integrity. Therefore, the observations obtained from histological sections and SEM images in this study should be interpreted as illustrative of general morphological outcomes and not as evidence of preservation of membrane functionality or ultrastructural stability.

Even though cell morphology appeared intact, the cryoprotective solutions used, based exclusively on MeOH or Me_2_SO as permeable cryoprotective agents, proved inadequate to support oocyte development. Notably, treatments containing high concentrations of MeOH (3.1 M) or Me_2_SO (1.3 M) did not result in morphologically viable embryos, suggesting cytotoxic effects at these levels, as reported in previous studies. The combination of high cryoprotectant concentration with prolonged equilibration time, as applied in this study, appears particularly detrimental to oocytes physiology. A similar finding was reported by Zampolla et al. (2009), who observed that MeOH concentrations between 3 and 4 mol/L led to reduced mitochondrial distribution in zebrafish oocytes. Similarly, Me_2_SO at concentrations of 2-4 mol/L caused immediate ATP depletion and impaired mitochondrial DNA maintenance, reinforcing the observed loss of viability.

Previous studies with zebrafish ovarian follicles showed that medium containing 50% L-15 was more effective than HBSS or KCl buffer in preserving cellular integrity after exposure to subzero temperatures (Zampolla et al., 2008; Tsai et al., 2009). In contrast, in the present study, the HBSS medium proved more suitable than the medium containing 50% L-15 for maintaining the viability of *P. brachypomus* oocytes. A possible explanation for this difference lies in the osmotic characteristics of the media.

Despite the trials were conducted independently, the current findings are consistent with those reported by Ahammad et al. (2003), who observed that embryos at more advanced developmental stages are more sensitive to cryopreservation than those at intermediate stages. In the present study, embryos subjected to Protocol 2 (P2E) were rendered nonviable after warming, likely due to insufficient dehydration and intracellular ice formation.

This result may be attributed, at least in part, to insufficient dehydration associated with the faster cooling rate applied in P2E, in compounded by the cryoprotectant solution used. Effective cryopreservation depends on the ability of the cell to lose water before intracellular freezing occurs. Optimal cooling rates are strongly influenced by cellular properties, as described by Mazur (1984) and Fuller (2004), including cell volume, membrane surface area and permeability to water and cryoprotectants. Fish embryos, characterized by large size, substantial yolk content and inherently low membrane permeability, are particularly susceptible to incomplete dehydration during rapid cooling. Under these conditions intracellular ice formation, is more likely to occur, representing one of the main causes of cryoinjury and post-thaw viability loss (Zhang and Rawson, 1995; Diwan et al., 2020).

The gradual cooling approach employed in Protocol 1 (P1E), with a 10-min equilibration period for each 10 °C reduction in temperature, was designed to facilitate the diffusion of the cryoprotectants into the cells and promote intracellular water efflux, thus reducing the risk of intracellular ice formation. However, the proportion of morphologically viable embryos after thawing remained low. This limited success may be related to the exclusive use of MeOH as a cryoprotectant, which appears insufficient to provide adequate protection under the tested conditions, a results comparable to the low viability observed in oocyte cryopreservation protocols based solely on MeOH.

Among the treatments tested in P1E, the SC5 cryoprotectant solution was the most effective among those evaluated, yielding 15.3% morphologically viable embryos after thawing. Notably, all solutions containing sucrose resulted in some degree of embryonic survival, whereas those incorporating polyvinylpyrrolidone (PVP) failed to produce any viable embryos. This observation align with previous findings by Dumoulin et al. (1994), who reported that PVP is less effective in preventing cryoinjury compared to other macromolecules. Although polymer-based cryoprotectants can enhance cell survival in certain contexts, PVP specifically has been linked to reduced post-thaw viability. In contrast, the combination of MeOH and sucrose has shown favorable results in other fish species, including *Cyprinus carpio* (Ahammad et al., 2003), zebrafish (Kopeika et al., 2006) and *Prochilodus lineatus* (Machado et al., 2019).

Sucrose contributes to cryoprotection by promoting osmotic dehydration and cell shrinkage, thereby minimizing the formation of intracellular ice crystals (Diwan et al., 2020). Methanol, due to its low molecular weight (32 g/mol), exhibits high membrane permeability. Because membrane transport and osmoregulation are central factors in cryodamage in embryonic cells, the combined use of MeOH and sucrose has been proposed as a suitable cryoprotective strategy for fish embryos, which are characterized by multiple compartments, high yolk content and inherently low membrane permeability.

Cell survival during cryopreservation is closely associated with membrane integrity, as the plasma membrane acts as a semipermeable barrier between the cytoplasm and the external environment. In the present study, frozen embryos may have experienced membrane-related damage resulting from the osmotic stress and uncontrolled water influx during the cooling process, as described for other biological system (Muldrew and Mcgann, 1994). However, it is important to emphasize that such damage could no be directly assessed by the histological and scanning electron microscopy (SEM) analyses performed. Membrane susceptibility to cryoinjury is also influenced by lipid composition, which affects key biochemical and biophysical properties, including resistance to thermal stress (Hayward et al., 2014). The presence of lipids, both as components of membrane architecture and as intracellular lipid droplets, has been identified as a major limiting factor for cryopreservation efficiency in several species, including fish (Isayeva et al., 2004). Even though, SEM images in the present study revealed structural disorganization in the cryopreserved embryos, particularly in the blastoderm region, these observations should be interpreted as qualitative indicators of morphological alteration rather than confirmation of specific molecular or cytoskeletal damage.

Intracytoplasmic lipid droplets are know to interact with intermediate filaments from the cytoskeleton (Okada et al., 2014) and such interaction have been associated with the maintenance of cell morphology (Bodin et al., 2005). Previous studies suggest that structural alterations in these domains can modify the physicochemical properties of the intracellular environment and disruption cytoskeleton organization (Isachenko et al., 2001), potentially leading to chromosomal abnormalities and defective cytokinesis, (Eroglu et al., 1998). In addition to structural injury, cryoprotectants themselves may alter gene expression patterns in embryos and larvae, as reported by (Zhang et al., 2021). These mechanisms were not directly evaluated in the present study and are discussed here as plausible explanations based on established cryobiological literature.

Notwithstanding previous studies have reported that *P. brachypomus* embryos can be preserved under cold storage condictions, such as at −10 °C for 6 h, 8 or 13 h post-fertilization (Pessoa et al., 2015). These protocols involved substantially less severe thermal exposure than the freezing conditions evaluated in the present study. Similar autcomes under moderate cooling have also reported for *Rhinelepis aspera*, *P. mesopotamicus* and *Salminus brasiliensis* using comparable cryoprotectants (Fornari et al., 2012, 2013, 2014). In contrast, the present study focused on more rigorous cryopreservation protocols intended for long-term storage, which expose embryos to greater osmotic and thermal stress. This methodological gap highlights the technical and biological challenges associated with transitioning from short-term cooling to effective cryogenic preservation, particularly in Neotropical fish species.

## Conclusion

The cryopreservation protocols tested for *P. brachypomus* oocytes and embryos demonstrated limited efficacy, as no successful post-thaw fertilization was achieved and embryo survival rates remained low. Among the treatments evaluated, the protocol combining MeOH and sucrose (SC5), when applied to embryos, yielded the highest proportion of morphologically viable embryos (15.3%), suggesting potential for further refinement. The data highlight the cytotoxic effects of high concentrations of cryoprotectants and the structural challenges imposed by fish embryos. Despite these limitations, the partial success observed provides a valuable foundation for refining cryopreservation strategies and advancing germplasm conservation efforts in Neotropical fish species.

## Data Availability

Research data is only available upon request.
